# Supplementary material for: Effect of 433 MHz double-slot microwave antennas for double-zone ablation in ex vivo swine liver experiment
Source: PLoS One. 2025 Feb 11;20(2):e0315678. doi: 10.1371/journal.pone.0315678 (PMC11813083; doi:10.1371/journal.pone.0315678)
Supplement: Attachment — Submitted filename: PONE-D-24-27447_review.docx [file pone.0315678.s002.docx]

This study provides a foundation for the development of advanced MWA techniques that can be tailored to individual patient needs, potentially improving outcomes for patients with complex tumor geometries. (major revision)

1. The objective is clearly stated in the 'Purpose' section of the abstract; however, the conclusion could more effectively emphasize the practical applicability of this research. For example, it could better connect the results to potential clinical applications beyond cancer therapies.
2. The terms describing ablation zones such as “gourd shape” or “guitar shape“, should be frequent and used consistently throughout the manuscript. And if this terminology does only first appear in the results, then a short description should be delivered already at an earlier stage of the Methods chapter.
3. The details of the ex vivo experiment might be more elegant. Essential experimental controls and the ways by which they were maintained should have been better detailed, especially on how temperature control of liver samples was achieved before ablation.
4. It should be justified on why the particular type of statistical tests were performed and how to interpret them. What, for example, was the specific quantification of those differences between experiment and simulation?
5. How antenna performance effect on whole experiment and please provide previous study antenna comparison according to the experiment.
